# Possible Mechanisms of the Neuroprotective Actions of Date Palm Fruits Aqueous Extracts against Valproic Acid-Induced Autism in Rats

**DOI:** 10.3390/cimb45020105

**Published:** 2023-02-14

**Authors:** Abdelaziz M. Hussein, Seham Ahmed Mahmoud, Khalid Mohammed Elazab, Ahmed F. Abouelnaga, Marwa Abass, Ahmed A. H. Mosa, Mennatullah A. M. Hussein, Mohamed E. G. Elsayed

**Affiliations:** 1 Department of Medical Physiology, Faculty of Medicine, Mansoura University, Mansoura 35516, Egypt; 2 Chemistry Department, Zagazig University, Zagazig 44519, Egypt; 3 Department of Biology, Faculty of Science, Jazan University, Jazan 45142, Saudi Arabia; 4 Department of Animal Husbandry and Development of Animal Wealth, Faculty of Veterinary Medicine, Mansoura University, Mansoura 35516, Egypt; 5 Department of Surgery, Anesthesiology and Radiology, Faculty of Veterinary Medicine, Mansoura University, Mansoura 35516, Egypt; 6 Department of Neurology, Faculty of Medicine, Delta University for Science and Technology, Gamasa 11152, Egypt; 7 Faculty of Medicine, Mansoura University, Mansoura 35516, Egypt; 8 Department of Psychiatry and Psychotherapy III, University of Ulm, 89075 Ulm, Germany; 9 Department of Psychiatry, School of Medicine and Health Sciences, Carl von Ossietzky University Oldenburg, 26129 Oldenburg, Germany

**Keywords:** palm date extracts, VPA, autism, Sirt-1, oxidative stress, Nrf2, Sirt-1 and LC3

## Abstract

The current study aimed to determine how palm date aqueous fruit extracts (AFE) affected the autistic-like behaviors brought on by valproic acid (VPA) injection, as well as any potential contributions from Sirt-1, oxidative stress, apoptosis, and autophagy. The pregnant Sprague Dawley females were treated with VPA at 12.5th gestation day and pregnant females and their offspring were treated with AFE orally at doses of 4 mg/Kg by gastric gavage for 45 days after birth. The elevated plus-T maze, water maze, and rotarod tests were used to examine autism-like behaviors. At the end of the study, the expression of Nrf2, heme oxygenase (HO-1), Sirt-1, caspase-3 (a marker of apoptosis), LC3 (a marker of autophagy), and NFκB (inflammatory cytokines) were evaluated along with the oxidative stress in brain tissues and the histological changes in the cerebellum and hippocampus. The neurobehavioral assessments significantly declined due to VPA, which also significantly increased oxidative stress in the brain tissues and significantly decreased Nrf2 and HO-1 expression. Additionally, VPA administration caused significant increase in the expression of caspase-3 in the cerebellar cortex, not in the hippocampus; LC3 and NFκB in the hippocampus, not in the cerebellar cortex; and significant reduction in the expression of Sirt-1 in the hippocampus, not in the cerebellum. On the other hand, AFE treatment significantly improved the neurobehavioral changes as well as it improved significantly the oxidative stress and the expression of LC3, NFκB, NrF2, HO-1, and Sirt-1 in the cerebellum and hippocampus. Conclusions: AFE administration might improve the autistic-like symptoms induced by VPA in rats via attenuation of the oxidative stress, upregulation of Nrf2 and HO-1, Sirt-1 and LC3 expression with downregulation of caspase-3, and NFκB expression in the cerebellum and hippocampus.

## 1. Background

A variety of behavioral abnormalities, including social impairment, poor communication abilities, and repetitive behavior characterize the neurological condition known as an autism spectrum disorder (ASD). According to Young et al. [1] and Mohammadi et al. [2], the prevalence of ASD among children ages 0 to 15 is around 0.11% and 0.09% in rural and urban settings, respectively [3]. As genetics and the environment significantly impact how the brain develops normally, behavioral disorders may also result from a combination of environmental and genetic factors. Even though the pathophysiology of ASD is not fully known, it is widely believed that genetic and environmental variables are crucial in the development of ASD and abnormal pre- and postnatal brain development [4,5]. Treatment of ASD is still challenging, though, as a result of its late diagnosis and the belief that any minor alteration to the nervous system during the early stages of development will be irreversible.

Numerous animal models of autism exist, which can be categorized into single-gene mutations, epigenetic factors/prenatal chemical exposure, neo-natal lesions of particular brain regions, and other genetic disorders connected to autism [6]. The administration of valproic acid (VPA), a gamma-aminobutyric acid (GABA) agonist anti-epileptic drug, to the pregnant rodent mothers at a crucial stage of gestation is a widely used animal model of autism spectrum disorder (ASD) [4]. This model was developed as a result of pharmaco-epidemiological research showing a strong correlation between maternal use of VPA during pregnancy (for example, to treat migraines, mania, or epilepsy) and the child’s later development of ASD [7,8]. It has been noted that offspring of rats treated to a single dosage of VPA on an embryonic day 12.5, just before neural tube closure and the creation of the brain stem nuclei in rats, develop neuroanatomical and behavioral traits resembling those of autistic people [9,10]. In particular, VPA-exposed offspring exhibit (1) a decreased number of motor cells in cranial nerve motor nuclei in the brain stem [11]; (2) a decreased number of Purkinje cells in the cerebellum, which has been seen in both autism [12] and offspring treated with VPA [13]; and (3) a decrease in social interactions, an increase in repetitive behaviors, and a decrease in pain sensitivity [14]. Along with enhanced synaptic plasticity in the amygdala, rat pups treated at the embryonic (E) 12.5th day also show highly amplified conditioned fear memories that generalize to non-conditioned stimuli and are resistant to extinction [15]. Autism is also frequently associated with abnormally high levels of anxiety and phobias [16]. Similar outcomes have been attained in rats [10] and mice using slightly modified VPA treatment methods [17]. The VPA rat model is well adapted to explore synaptic and circuit abnormalities in order to clarify the probable neuropathology of autism given these significant behavioral and anatomical similarities between the rat model and human autism.

Growing research has demonstrated that compounds with antioxidant and neuroprotective properties, such as *Bacopa monnieri* plant extracts [18], resveratrol [19], green tea [20], and piperine [21] can reduce autism-like behavior induced by valproic acid (VPA) [22]. Therefore, we hypothesized that a substance possessing antioxidant and neuroprotective effects might provide benefits for patients with autism. Due to the high concentrations of phenolic compounds, flavonoids, anthocyanins, coumaric acid, and ferulic acid in date palm (*Phoenix dactylifera* L.), it possesses potent antioxidant properties [23]. Date palm fruit and seed extracts have been shown to have potent antioxidant effects in several in vitro [24,25] and in vivo studies (reviewed in [26]). Furthermore, El-Mousalamy et al. [27] concluded that date palm fruit and seed extracts, both aqueous and methanolic, protect rats’ kidneys from diabetic nephropathy by acting as antioxidants. Additionally, a recent study conducted by our research team found that date palm extracts had renoprotective effects against renal ischemia/reperfusion (I/R) injury, which may have been caused by an increase in the expression of the transcription factor Nrf2, as well as a decrease in oxidative stress, apoptosis, and inflammatory cytokines (TNF-α and TGF-β) [28]. Therefore, the current study aimed to investigate the potential neuroprotective effects of aqueous palm date fruit extracts (AFE) against VPA-induced autistic disease in rats, as well as the possible role of oxidative stress, antioxidant genes (Nrf2 and HO-1), apoptosis (via caspase-3 expression), autophagy (LC3 expression), the inflammatory process (NFκB), and Sirt-1 protein in the cerebellum and hippocampus.

## 2. Materials and Methods

### 2.1. Preparation of Date Palm Fruit Aqueous Extract

We extracted 100 g of the freshly blended fruits in one liter of distilled water, and the mixture was sieved and evaporated to obtain the crude extract. Details of the extraction process were mentioned in a previous study by our group [28]. HPLC analyses of the fruit aqueous extracts revealed that it contains 8 compounds from highest concentration to the lowest: p-coumaric acid, caffeic acid, ferulic acid, chlorogenic acid, sinapic acid, quercitin-3-o-glycoside, apiginin-c-glycoside, and luteoline-7-o-glycoside [27].

### 2.2. Experimental Protocol

All experimental protocols and procedures were done according to the guidelines of animal care and use and approved from the Animal Care and Use Committee, Mansoura University (code # MU-ACUC VM.R.22.10.11). Sprague Dawley female rats were purchased from the Medical Experimental Research Center (MERC) and housed in the Research Facility of Mansoura Faculty of Veterinary Medicine at a 12 h light/dark cycle. Rats were divided into 4 groups as follows;

1. Group I. Naïve control: the offspring male rats in this group received no treatment.
2. Group II. Naïve control + AFE: the mothers (from embryonic E12.5th day until labor) and offspring male rats in this group were treated with aqueous fruit extracts (AFE) (4 mg/kg dissolved in 1 mL saline/day via gastric gavage) until PND45 [28].
3. Group III. VPA group: the pregnant female rats in this group received single intraperitoneal dose of valproic acid (VPA) (500 mg/Kg) at E12.5th embryonic day
4. Group IV. VPA + AFE: the offspring male rats in these groups were treated with VPA (500 mg/kg single i.p. injection at day 12.5) and aqueous extracts of palm date fruits to the mother (4 mg/kg dissolved in 1 mL saline/day via gastric gavage) until the time of labor and to the offspring until PND45 [28].

### 2.3. Behavioral Tests

#### Rotarod Test

Each animal was placed on a rod rotating at a speed of 40 revolutions per minute (rpm) on postnatal day (PND) 21 and PND45. The time taken by each animal to maintain its balance on the rod within a 5 min period was recorded as the endurance time [29].

### 2.4. Elevated Plus-Maze Test

Although anxiety is not the primary clinical manifestation of autism, anxiety is a real difficulty for patients with autism [30]. Therefore, we also evaluated anxiety by using the elevated plus-maze test. All animals were directly placed on the elevated plus maze consisting of two open and two closed arms (50 cm length, 12 cm wide, 30 cm high) and placed approximately 50 cm from the floor. Each rat was placed in the center of the maze facing an open arm and was allowed to freely explore for 5 min. The percent of time spent in the open arm and time spent in entering the open arm were recorded [31].

### 2.5. Morris Water Maze Test

The effect of AFE on memory was evaluated based on the previous finding that autism patients often display a memory deficit [32]. The animals were exposed to a pool of 4 quadrants (Northeast, Southeast, Southwest, and Northwest) filled with tap water (25 °C, 40 cm deep) and covered with nontoxic milk. The removable platform was immersed below the water surface of one quadrant. Each animal had to memorize the location of the immersed platform by using specific visual cues placed around the outside of the tank. The time each rat spent finding and climbing on the immersed platform was recorded as the escape latency. Twenty-four hours later, the animal was re-exposed to the same condition, except that the immersed platform had been removed. Each rat’s mean time in the target quadrant to search for the missing platform was noted as the retention time and was used as the index of retrieval memory. The escape latency and the retention time in a 5 min exposure time were used as indices of learning and memory. A blind observer always stood in the same position, and care was taken not to disturb the relative location of the water maze concerning other objects in the laboratory. The spatial memory capacity was determined via this test on PND21 and PND45 [29].

### 2.6. Harvesting of Brain Tissues

Details of the collection and storage of brain tissues are described in our previous work [33]. Briefly, by the end of the study at PND45, the rats were sacrificed by a high dose of Na^+^ thiopental (120 mg/kg) i.p. For histological and immunostaining studies, the brain was perfused with 100 mL heparinized saline and then by 150 mL formalin (10%) through a cardiac catheter and the brain tissues were stored in formalin (10%). However, the brain tissues for biochemical and molecular studies were only collected after perfusion with saline and stored at –80 °C for further investigation.

### 2.7. Assay of Oxidative Stress Markers (MDA, Catalase Activity, and Total Antioxidants) in Brain Tissues 

Hippocampal regions of the brain and cerebellum were homogenized in 1–2 mL cold phosphate-buffered saline (50 mM) in EDTA (1 mM) at pH 7.5, then centrifuged at 950× *g* at 4 °C for 15 min. Colorimetric assay of these markers was done by using commercially available kits (Bio-Diagnostics, Dokki, Giza, Egypt) according to the manufacturer’s instructions.

### 2.8. Assessment of the Expression of the Antioxidant Transcription Factor (Nrf2) and HO-1 at the Level of mRNA by Real-Time PCR

mRNA encoding for an antioxidant transcription factor; Nrf2; and heme oxygenase (HO)-1 was identified by real-time PCR in brain tissues. According to the manufacturer’s instructions, we isolated the total RNA from brain tissue specimens using RNA extraction kits (Invitrogen Corporation, Grand Island, NY, USA). RNA was quantified spectrophotometrically, and its quality was determined by agarose gel electrophoresis and ethidium bromide staining. Reverse transcription was done using 1 µg total RNA in 10 µL and equal volume of RT mix buffer (high-capacity cDNA reverse transcription kits (applied biosystem, cat # 4374966) with total reaction volume of 20 µL. All gene details (tested genes including Nrf2 and HO-1, and house-keeping gene, GAPDH) amplification and detection were mentioned in our previous work [28].

### 2.9. Hematoxylin and Eosin Staining

Staining of brain tissues with hematoxylin and eosin (H&E) was done according to our previous work [33]. The hippocampal CA3 and CA1 regions and cerebellum were examined under a light microscope. Hippocampal regions were examined for the density of pyramidal cells, while the cerebellum was examined for the density of Purkinje cells. The number of Purkinje cells (PC) was counted in five sections of the cerebellum at magnification of 400× (high power field) in five rats at least, and the mean was calculated [34].

### 2.10. Immunohistochemical Examination for NF-κB, Caspase 3, and Sirt-1, and LC3 in CA3 and CA1 Regions of the Hippocampus and Cerebellum

Serial sagittal sections (40 µm) were obtained from brain tissues, and immunohistochemical staining was performed according to previous work [33]. The primary rabbit polyclonal anti-*NFκB*-p65 (Santa Cruz Biotechnology Inc., Santa Cruz, CA, USA) (with final dilution 1:500), goat polyclonal anti-Sirt-1 (Santa Cruz sc-19857, Santa Cruz, CA, USA) (with final dilution 1:200), caspase-3 primary rabbit polyclonal anti-caspase-3 (Cell Signalling Technology^®^ Cat. no. 9664, Danvers, MA, USA) (diluted 1:50) were used. The hippocampus and cerebellum were examined using an Optika digital camera, and the CA3 and CA3 hippocampal regions were analyzed for the immunostaining of these markers. The average number of positive cells for caspase-3, LC3, Sirt-1, and NFκB in 5 high-power fields (HPF) in 5 sections was calculated.

### 2.11. Statistical Analysis

Statistical analysis was done by GraphPad Prism version 5.0. One-way ANOVA with Tukey’s post-hoc test was used to find the statistical significance in biochemical, molecular, and histochemical parameters. Data were considered statistically significant when *p* ≤ 0.05.

## 3. Results

### 3.1. Effects of AFE on Spatial Memory Deficits and Motor Coordination in VPA-Induced Autism 

**Figure 1:**
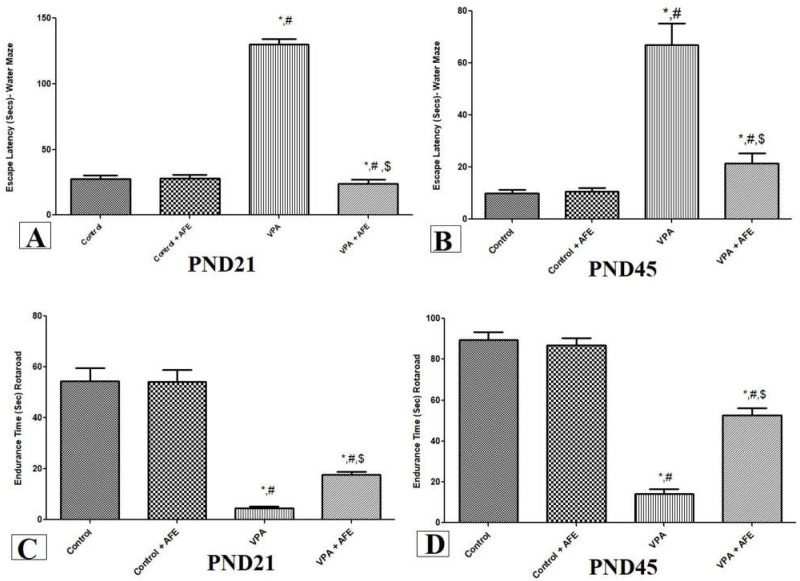
Effect of AFE on spatial memory and motor coordination evaluated by using the water maze and rotarod tests, respectively. The effect of AFE on the escape latency (secs) at PND21 (**A**) and PND 45 (**B**), and the effect of AFE on endurance time at PND21 (**C**) and PND45 (**D**). Data are expressed as mean ± SEM. * significant vs. control group, # significant vs. control + AFE group, $ significant vs. VPA group. *p* ≤ 0.05 is considered significant.

The results of the Morris water maze test’s escape delay are shown in Figure 1A,B. In comparison to the control and AFE groups, the escape delay of the rats in the VPA group was considerably higher on PND21 and PN45 (*p* < 0.001). On the other hand, the VPA + AFE group significantly reduced the escape latency at PND21 and PND45 in comparison to the VPA group (*p* < 0.01).

Figure 1C,D depicts the results of the rotarod test, which was used to evaluate the impact of AFE on motor coordination at PND 21 and PND 45. According to the findings, rats given VPA treatment had significantly shorter endurance times at PND 21 and PND 45 compared to the control and AFE groups (*p* < 0.001). On the other hand, the VPA + AFE group’s endurance time was considerably longer than the VPA group’s at PND21 and PND45 (*p* < 0.01).

### 3.2. Effects of AFE on Anxiety in VPA-Induced Autism 

**Figure 2:**
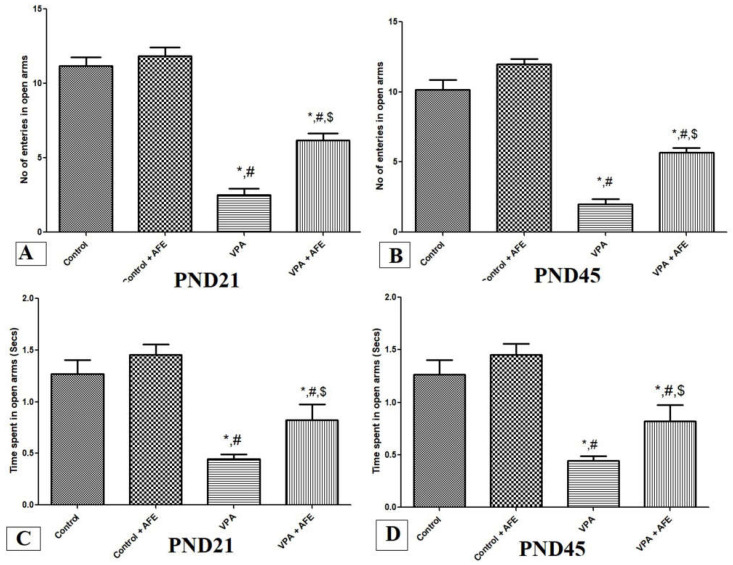
Effect of AFE on anxiety evaluated by using the elevated plus- maze. The effect of AFE on the number of entries on open arms at PND21 (**A**) and PND 45 (**B**) and the effect of AFE on time spent (secs) in open arms at PND21 (**C**) and PND45 (**D**). Data are expressed as mean ± SEM. * significant vs. control group, # significant vs. control + AFE group, $ significant vs. VPA group. *p* ≤ 0.05 is considered significant.

The elevated plus-maze was used at PND21 and PND45 to gauge how AFE affected anxiety. The findings showed that, at PND21 and PND45, the number of open-arm entrances and the amount of time spent in open arms in the VPA group were significantly lower than those in the control and AFE groups (*p* < 0.001). On the other hand, at PND21 and PND45, the VPA + AFE group significantly outperformed the VPA group in terms of both the number of entries and the amount of time spent in open arms (*p* < 0.01) (Figure 2A–D).

### 3.3. Effects of AFE on Oxidative Stress Markers (MDA and Catalase) and Expression of Nrf2 and HO-1 in VPA-Induced Autism

**Figure 3:**
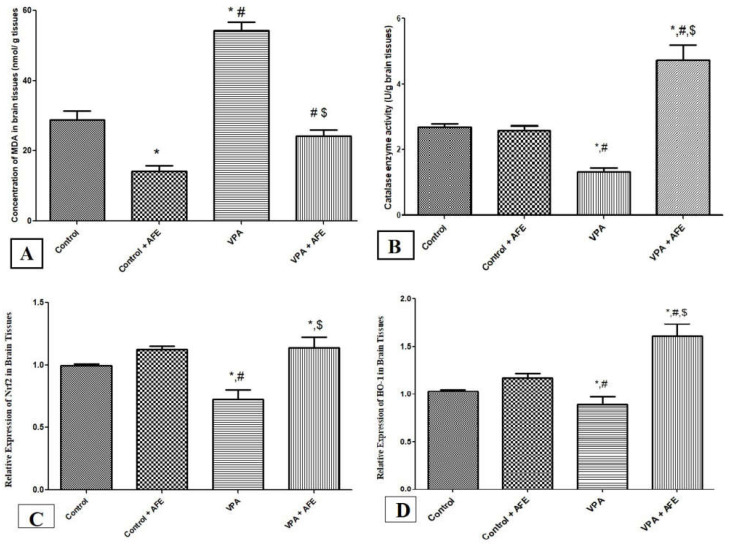
Effect of AFE on oxidative stress markers (MDA and catalase) and the expression of antioxidant genes (Nrf2 and HO-1). The effect of AFE on the concentration of MDA (marker of lipid peroxidation) (**A**), catalase enzyme (antioxidant enzyme) (**B**), expression of Nrf2 at mRNA (**C**) and expression of HO-1 at mRNA levels (**D**). Data are expressed as mean ± SEM. * significant vs. control group, # significant vs. control + AFE group, $ significant vs. VPA group. *p* ≤ 0.05 is considered significant.

While catalase activity does not substantially differ between the control and AFE groups, the concentration of MDA in the brain tissues was significantly lower in the AFE group compared to the control group (*p* < 0.05). However, compared to the control and AFE groups, the MDA concentration in the VPA group was significantly higher (*p* < 0.001), and the catalase activity in the VPA group was significantly lower (*p* < 0.001). Additionally, the catalase activity was significantly higher in the VPA + AFE group compared to the VPA group (*p* < 0.001), whereas the concentration of MDA was significantly lower in the VPA + AFE group ((*p* < 0.001) (Figure 3A,B). In comparison to the control and AFE groups, the expression of Nrf2 and HO-1 at the mRNA level was considerably lower in the VPA group (*p* < 0.01). However, when compared to the VPA group, their expression levels were significantly higher in the VPA + AFE group (*p* < 0.01) (Figure 3C,D).

### 3.4. Effects of AFE on Cerebellar and Hippocampus Morphology in VPA-Induced Autism

**Figure 4:**
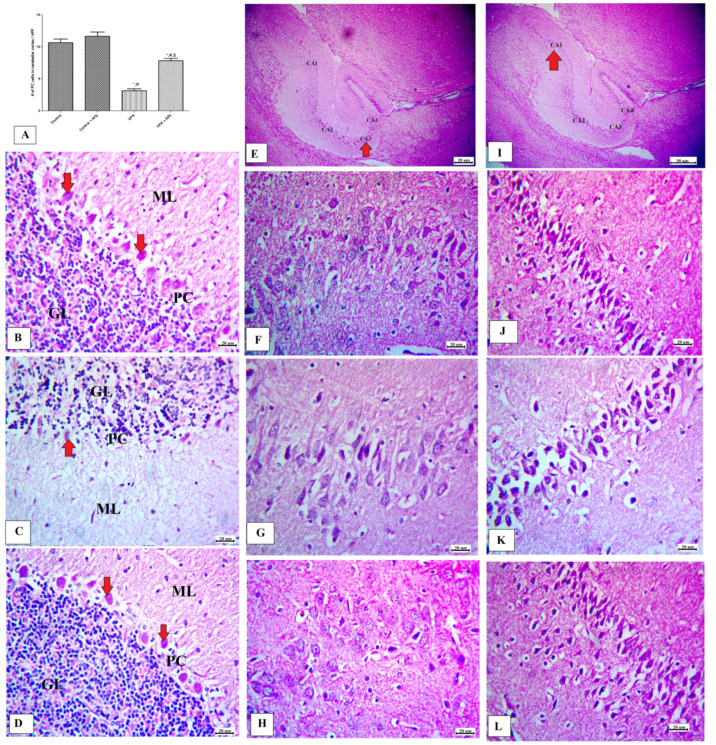
Effect of AFE on morphology of the cerebellum and hippocampus. (**A**) shows the mean number of Purkinje cells (PC) in the cerebellar cortex in different groups. The cerebellar cortex of the control group (**B**) consists of three layers; outer molecular layer (ML), middle Purkinje cell layer (PC), and inner granular cell layer (GL). VPA-treated group (**C**); PC appeared shrunken, disfigured with condensed chromatin (red arrows) and surrounded with vacuolated spaces which became few in number. The ML showed deeply stained pyknotic scattered basket cells and vacuolations. In the VPA + AFE group (**D**), the number of PC become high (400×, H&E). (**E**) shows different parts of hippocampus with red arrows indicating CA3 region (40×). (**F**–**H**) show CA3 regions in brain from control, VPA, and VPA + AFE groups, respectively (400×). (**I**) shows different parts of hippocampus with red arrows indicating CA1 region (40×). (**J**–**L**) show CA3 regions in brain from control, VPA, and VPA + AFE groups, respectively (400×). The number of Purkinje cells; (**D**) were dramatically decreased in VPA-treated group; * significant vs. control group, # significant vs. control + AFE group, and $ significant vs. VPA group.

The effects of AFE on the cerebellar cortex and hippocampal regions (CA1 and CA3) are shown in Figure 4. Figure 4A shows that VPA significantly decreased both the numbers and the sizes of Purkinje cells (PC) in the cerebellum, while treatment with AFE caused a significant rise in PC numbers in the cerebellar cortex (*p* < 0.01). Figure 4B–D represents cerebellum specimens from control, AFE, VPA, and VPA + AFE groups, respectively. The cerebellar cortex of the control group consists of three layers; the outer molecular layer (ML) (contains nerve fibers, stellate cells and basket cells), the middle Purkinje cell layer (PC) (single row of flask-shaped cells with large rounded nuclei), and the inner granular cell layer (GL) (contains granule nerve cells) (Figure 4B). Those obtained from VPA-treated group show shrunken, disfigured PC with condensed chromatin (thick white arrows) surrounded with vacuolated spaces, which and became few in number, and the ML showed deeply stained pyknotic scattered basket cells and vacuolations (Figure 4C). On the other hand, the AFE-treated group showed normal morphology of the cerebellar cortex (Figure 4D). Additionally, Figure 4E and I indicate the CA3 and CA1 regions of hippocampus (red arrow) at low power of the control group, respectively; while Figure 4F–H represents the CA3 region from the control group, VPA group (low number of normal nerve cells), and VPA + AFE group, respectively; and Figure 4J–L represents the CA1 region from the control group, VPA group (low number of normal nerve cells), and VPA + AFE group, respectively.

### 3.5. Effects of AFE on the Expression of Apoptotic Marker (Caspase-3), Sirt-1, Inflammatory Marker (NFκB), and Autophagic Marker (LC3) by Immunohistochemistry in Cerebellum and Hippocampus in VPA-Induced Autism

**Figure 5:**
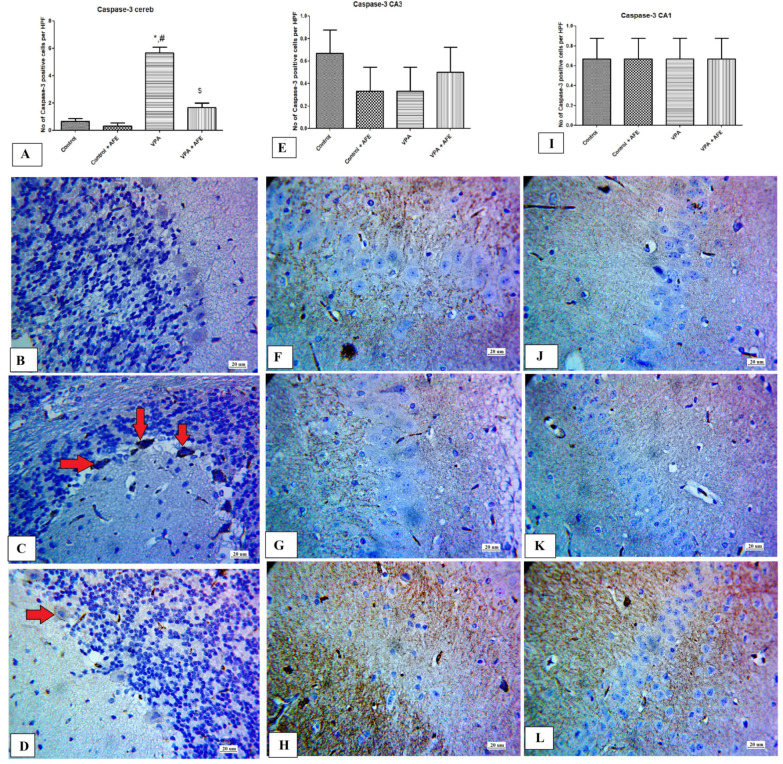
Effect of AFE on the caspase-3 expression in the cerebellum and hippocampus. (**A**,**E**,**I**) show the mean number of caspase-3 positive cells in the Purkinje cells (PC) layer of the cerebellar cortex, CA3 hippocampal region, and CA1 hippocampal region of different groups, respectively. (**B**–**D**) show cytoplasmic expression of caspase-3 (red arrows) in the PC of the cerebellar cortex of the control, VPA, and VPA + AFE groups, respectively. (**F**–**H**) show negative cytoplasmic expression of caspase-3 in CA3 of the control, VPA, and VPA + AFE groups, respectively. (**J**–**L**) show negative cytoplasmic expression of caspase-3 in CA1 of the control, VPA, and VPA + AFE groups, respectively (400×). * significant vs. control group, # significant vs. control + AFE group, and $ significant vs. VPA group.

In comparison to control and AFE groups, the expression of caspase-3 in Purkinje cells of the cerebellar cortex was considerably higher in the VPA group (*p* < 0.01) and significantly lower in the VPA + AFE group (*p* < 0.001) than in the VPA group. However, the expression of caspase-3 in the CA1 and CA3 hippocampal regions did not change significantly (Figure 5A,E,I). Figure 5B shows negative caspase-3 expression in the control and AFE groups, while Figure 5C shows moderate cytoplasmic expression of caspase-3 in PC of the cerebellar cortex in VPA group and minimal in the VPA + AFE group (Figure 5C,D). Figure 5F,G shows negative caspase-3 expression in in the CA3 region of the various study groups (control, AFE, VPA, and VPA + AFE group, respectively). Furthermore, Figure 5J–L shows negative caspase-3 expression in in the CA1 region of the various study groups (control, AFE, VPA, and VPA + AFE group, respectively).

**Figure 6:**
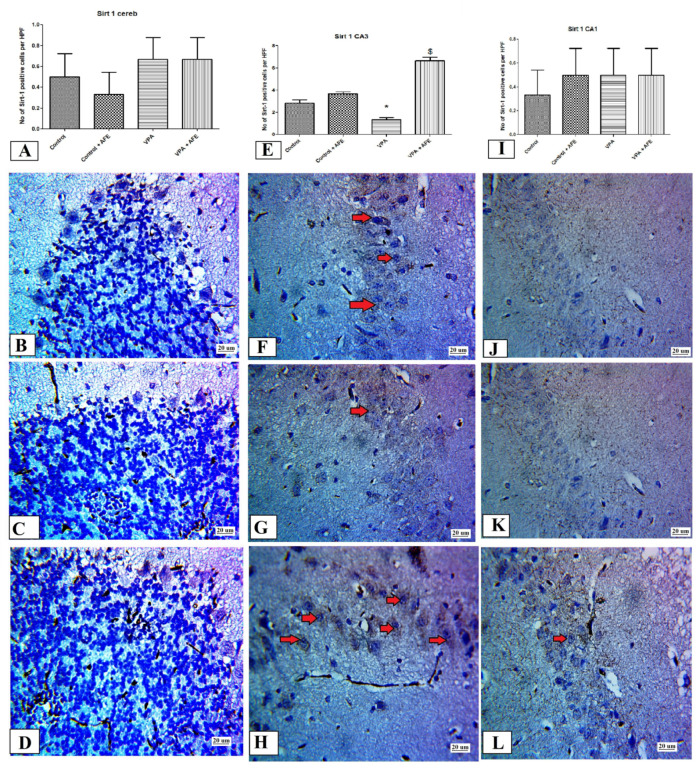
Effect of AFE on the Sirt-1 expression in the cerebellum and hippocampus. (**A**,**E**,**I**) show the mean number of sirt-1 positive cells in the Purkinje cells (PC) layer of the cerebellar cortex, CA3 hippocampal region, and CA1 hippocampal region of different groups, respectively. (**B**–**D**) show negative cytoplasmic expression of sirt-1 in the PC of the cerebellar cortex of the control, VPA, and VPA + AFE groups, respectively. (**F**–**H**) show positive cytoplasmic expression (red arrows) of sirt-1 in CA3 of the control (mild to moderate), VPA (minimal), and VPA + AFE groups (marked), respectively. (**J**–**L**) show negative cytoplasmic expression of Sirt-1 in CA1 of the control and VPA, and minimal cytoplasmic expression in the VPA + AFE group, respectively (400×). * significant vs. control group, and $ significant vs. VPA group.

Figure 6A shows no statistically significant difference for Sirt-1 expression in the cerebellar cortex among the different studied groups, while Figure 6E shows significant decrease in Sirt-1 expression in CA3 region of VPA group with significant increase in its expression in the VPA + AFE group compared to VPA groups (*p* < 0.01) and Figure 6F shows no statistically significant difference for Sirt-1 expression in CA1 among the different studied groups. Furthermore, in various study groups, the cerebellar cortex cell layers, including Purkinje cells, displayed negative immunoreactivity for Sirt-1 (Figure 6B–D). The CA3 hippocampal area, on the other hand, displayed little cytoplasmic expression for Sirt-1 in the VPA and control groups, and moderate cytoplasmic expression for Sirt-1 in the VPA + AFE groups (Figure 6F–H); it also displayed negative expression in CA1 in control group (Figure 6J) and VPA group (Figure 6K) and minimal expression in VPA + AFE group (Figure 6L).

**Figure 7:**
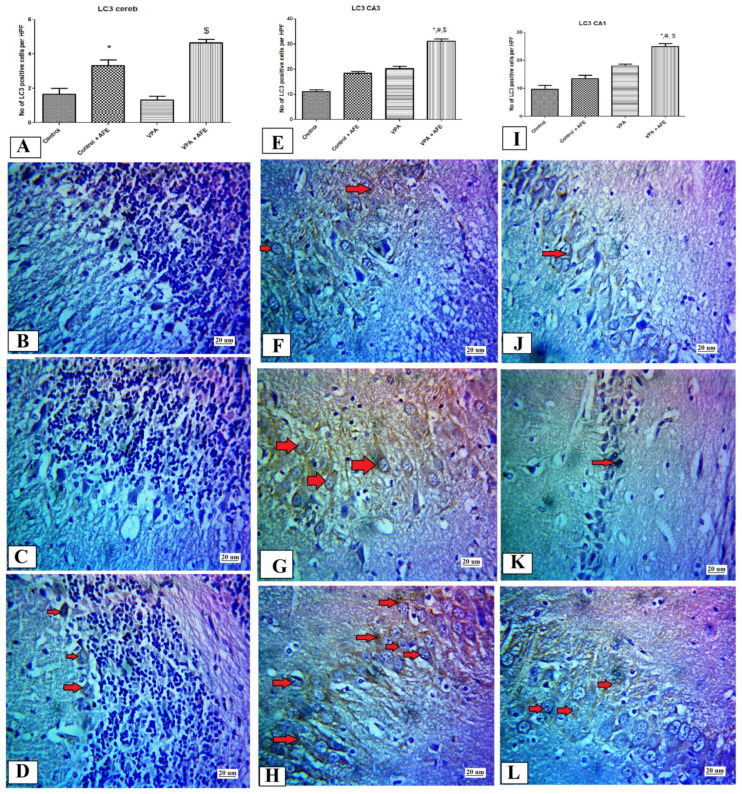
Effect of AFE on the LC3 expression in the cerebellum and hippocampus. (**A**,**E**,**I**) show the mean number of LC3-positive cells in the Purkinje cells (PC) layer of the cerebellar cortex, CA3 hippocampal region, and CA1 hippocampal region of different groups, respectively. (**B**) shows negative cytoplasmic expression of LC3 in the PC of the cerebellar cortex of the control, minimal LC3 expression in the VPA group (**C**), and moderate expression in the VPA + AFE group (**D**). (**F**–**H**) show positive cytoplasmic expression (red arrows) of LC3 in CA3 of the control group (mild to moderate), VPA group (mild-moderate), and VPA + AFE groups (marked), respectively. (**J**,**K**) show minimal cytoplasmic expression of LC3 in CA1 of the control group and VPA group, and (**L**) moderate cytoplasmic expression in the VPA + AFE groups, respectively (400×). * significant vs. control group, # significant vs. control + AFE group, and $ significant vs. VPA group.

LC3 expression in the cerebellar cortex shows a significant increase in the AFE group compared to the control group and in the AFE +VPA compared to the VPA group (*p* ˂ 0.01). While, in the CA3 and CA1 regions of the hippocampus, the expression of LC3 was significantly higher in the VPA + AFE group compared to the control and VPA groups (*p* < 0.01) (Figure 7A,E,I). In the control and AFE groups, the cerebellar cortical cell layers, including Purkinje cells, displayed low immunoreactivity in the control and VPA groups and moderate expression in the VPA + AFE group for LC3 (Figure 7B–D). Figure 7F–H,J–L shows cytoplasmic expression for LC3 in CA3 and CA1 hippocampal regions in the control, VPA, and VPA + AFE groups.

**Figure 8:**
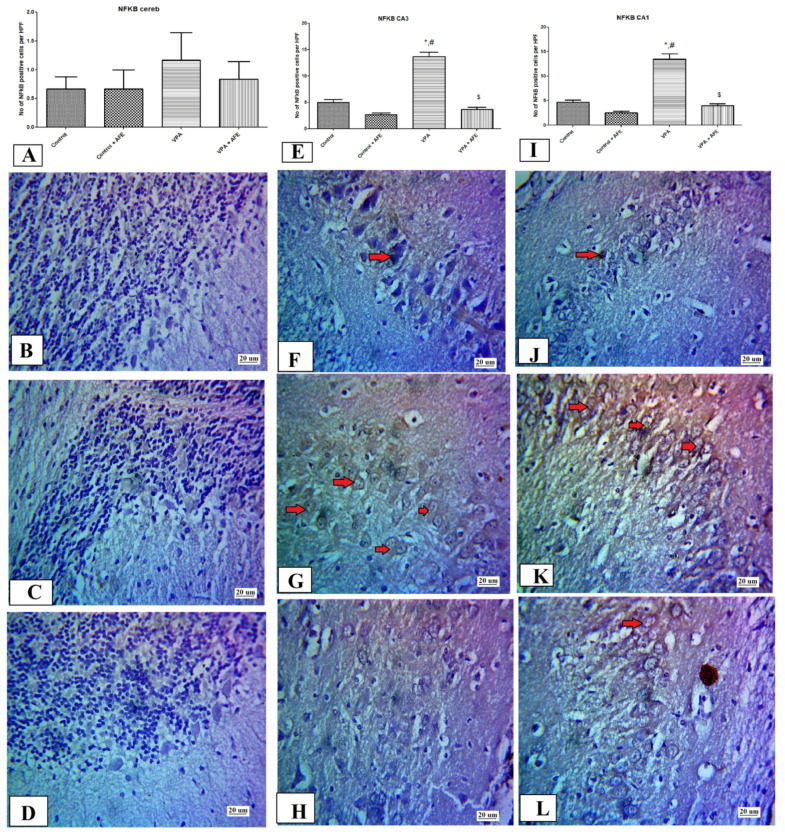
Effect of AFE on the NFκB expression in the cerebellum and hippocampus. (**A**,**E**,**I**) show the mean number of NFκB positive cells in the Purkinje cells (PC) layer of the cerebellar cortex, CA3 hippocampal region, and CA1 hippocampal region of different groups, respectively. (**B**–**D**) show negative nuclear and cytoplasmic expression of NFκB in the PC of the cerebellar cortex of the control, VPA group, and VPA + AFE group, respectively. (**F**–**H**) show positive nuclear expression (red arrows) of NFκB in CA3 of the control group (minimal), VPA group (mild–moderate), and VPA + AFE groups (minimal), respectively. (**J**–**L**) show nuclear expression of NFκB in CA1 of the control group and VPA group, and VPA + AFE groups, respectively (400×). * significant vs. control group, # significant vs. control + AFE group, and $ significant vs. VPA group.

The expression of NFκB in the cerebellar cortex shows no statistically significant difference among all studied groups, while its expression in the CA3 and CA1 regions of the hippocampus showed a significant increase in the VPA group compared to the control group (*p* < 0.01), with a significant decrease in its expression in the VPA + AFE group compared to the VPA group (*p* < 0.01) (Figure 8A,E,I). The cerebellar cortical cell layers, including Purkinje cells, displayed negative NF-κB immunoreactivity in the control, VPA, and VPA + AFE groups (Figure 8B–D). The CA3 and CA1 hippocampal areas of the VPA group displayed strong cytoplasmic expression for NF-κB (Figure 8G,K, respectively), while the control and VPA + AFE groups displayed little cytoplasmic expression of NF-κB (Figure 8B,H,J,L).

## 4. Discussion

In this study, we looked at how AFE affected cerebellar cortex and hippocampal morphology, as well as memory and motor deficits in a rat model of autism caused by VPA. In fact, the cerebellar hemisphere’s primary physiological function is regulating motor function, including movement, gait, and posture [35]. Additionally, it has been stated that the cerebellum collaborates with the prefrontal cortex, posterior parietal cortex, and cortical motor region to modulate cognitive function [36]. These processes can be disturbed by damage to the cerebellar cortex, which impairs motor, sensory, and memory abilities. Additionally, cerebellar injury altered the prefrontal cortex’s functionality, which is critical for social interaction [37]. According to the results of neurobehavioral tests conducted on the rats at days PND21 and PND45 of the current study, a single dose of VPA (500 mg/kg at E 12.5th day) significantly impaired their cognition, memory, and motor abilities. Morakotsriwan et al. [31], Schneider et al. [38], Schneider and Przewlocki [29], Kim et al. [10], and Zhang et al. [39] all reported similar findings. According to Morakotsriwan et al. [31], VPA injection (400 mg/Kg i.p.) at E 12.5th day causes autistic-like symptoms, including motor dysfunctions on the rotarod test at PND24, 25, 26, memory deficits on the Morris water maze at PND36–39, and anxiety on the elevated plus-maze (at PND30, 35, 40). These results also supported the development of autistic syndrome following VPA 500 mg/Kg dosing. The presence of cerebellar cortex injury was confirmed by histopathological examination of the brain, and the number of Purkinje cells was significantly decreased in the VPA-treated group compared to the control group of rats.

Contrarily, in the present work, we discovered that pretreatment with AFE significantly improved memory and motor deficits as well as anxiety in the offspring of mothers treated with VPA, indicating that palm date may play a neuroprotective role in the rat form of autism. In agreement with the findings of the current study, several studies demonstrated the beneficial effects of dates on neurological diseases such as Alzheimer’s disease (AD), Parkinson’s disease (PD), and Huntington’s disease (HD) and amyotrophic lateral sclerosis (ALS) [40,41]. Additionally, Li et al. [42] reported that regular consumption of these fruits is usually associated with a lower risk of neurodegenerative disorders and better cognitive performance in the elderly. Moreover, the aqueous date fruit extract has been shown to prevent neuronal circuitry against focal cerebral ischemia [43] and prevent neuronal necrosis in hypoperfused brains [44]. To the best of our knowledge, this discovery is the first to show that palm date extract protects against VPA-induced autism. These neuroprotective effects of palm date extracts could be due to its active ingredients including antioxidants such as p-coumaric acid, caffeic acid, ferulic acid, chlorogenic acid, and sinapic acid [27].

In this study, we did suggest that the decreased motor, sensory, learning, memory, and social behavior in VPA-treated rats may have been caused by increased oxidative stress [45], which in turn caused Purkinje cells in the cerebellum to degenerate, disrupting the corticocerebellar circuit. Previous research suggested that oxidative stress was a major factor in the development of neurodegeneration [45]. A study by Baldaçara et al. [46] also demonstrated that the cerebellum is essential for emotion regulation and that malfunction can result in a range of psychotic disorders. Additionally, previous studies provided strong evidence that oxidative stress plays a crucial role in autism by elevating LPO and lowering the brain’s serum level of antioxidant proteins [47]. In the current study, we found that there was a significant elevation the brain MDA (lipid peroxidation marker) concentration and a significant reduction in the catalase (antioxidant) enzyme activity in VPA-treated rats suggesting enhanced oxidative stress in the brain of VAP-induced autistic rats. Moreover, we found significant improvement in the oxidative stress in the brain of rats treated with AFE, suggesting an antioxidant effect for palm dates extracts in autistic rats. In line with the findings of the current study, Edobor, et al. [48] reported that *Pheonix dactylifera* fruits relieved the oxidative stress associated with several neurological disease conditions including neurodegeneration and movement disorders. However, to the best of our knowledge, this is the first study that reports the antioxidant effect of palm date extracts in the VPA-induced autism rat model. In addition, the current study reported a significant reduction in the expression of Nrf2 (antioxidant transcription factor) and heme oxygenase-1 in the brain of VPA-treated rats, suggesting that the endogenous antioxidant systems in the brain tissues of autistic rats might be impaired. Moreover, treatment with AFE significantly improved the expression of Nrf2 and HO-1, suggesting that upregulation of Nrf2 and HO-1 might be a potential mechanism for the neuroprotective effect of palm date extracts in autism. Similar findings are reported by previous studies, e.g., Pujari et al. [44] reported that the AFE attenuated oxidative stress and prevented neuronal necrosis in hypoperfused brains. On the other hand, Alghamdi et al. [28] reported that AFE caused a significant increase in Nrf2 in renal ischemia. Furthermore, Sandhya et al. [18] reported the same effect for *Bacopa monniera* (L.) Wettst, a medicinal plant traditional used as neurotonic agents against VPA-induced autism. They concluded that *B. monniera* improved significantly the behavioral alterations and autistic symptoms via attenuation of oxidative stress markers.

Additionally, oxidative stress plays a crucial role in cell apoptosis, and previous studies in mice suggested that exposure to VPA resulted in elevated expression of apoptotic markers that have been noticed in the neuroepithelium and are hypothesized to be the critical cause of neural tube defects in the offspring and changed embryonic signaling pathways [47]. So, in the current study, we examined the expression of caspase-3 (apoptotic marker) in the cerebellum and hippocampus of VPA-treated rats and found that there was no significant increase in its expression in hippocampal regions, but its expression was increased significantly in Purkinje cells of the cerebellar cortex. These findings suggest the involvement of cerebellar cortical apoptosis in autistic-like behavioral changes. Moreover, we found that AFE treatment caused significant attenuation of elevated caspase-3 expression, suggesting the anti-apoptotic action of AFE in VPA-induced autism. Alghamdi et al. [28] reported anti-apoptotic action for AFE in renal ischemia.

In various animal models of autism, neuroinflammation and immune-linked malfunctions have been observed [49]. The pathophysiology of ASD is linked to immunological and inflammatory changes by growing clinical and experimental evidence. According to the study’s findings [50], autistic people had higher amounts of pro-inflammatory cytokines and autoantibody synthesis in their brains. Studies on patients revealed an aggressive neuroinflammatory response with pronounced astroglial and microglial activation. The two cytokines that this neuroglia emitted that were the most prevalent in the brain tissues were tumor growth factor (TGF)-β1 and macrophage chemoattractant protein (MCP)-1 [51]. According to a study, greater expression of adhesion molecules such as neutrophil CD11b and endothelium ICAM-1 is linked to increased leukocyte–endothelial adhesion in animal models [52]. By increasing neutrophil cathepsin B levels, VPA improves leukocyte–endothelial adhesion in the cerebral arteries of autistic people. The level of the endothelium chemokine CXCL7 increases in conjunction with these molecular activations [47]. In the current study, we found that administration of VPA caused significant increase in NFκB in hippocampal regions (CA1 and CA3) not in the cerebellar cortex and AFE treatment caused a significant attenuation of this upregulation of NFκB. In line with these findings, the anti-inflammatory action of AFE was documented in previous studies of Alzheimer’s diseases animal models (reduces pro-inflammatory cytokines such as IL-1β, IL-2–6, IL-9,10, TNF-α, and eotaxin activity) [53] and renal ischemia animal models (reduces TNF-α and TGF-β) [28].

In the current study, we hypothesized that autophagy could play a role in VPA-induced autism. Previous studies reported that mTOR inhibition exhibited more autophagosomes than the VPA group, along with a greater LC3 II/LC3 I ratio and Bcl-2 expression [47]. In the current work, we found that VPA caused significant increase in the expression of autophagic marker (LC3) in hippocampal regions, not the cerebellar cortex. Moreover, treatment with AFE caused more significant increase the autophagic marker LC3 in the hippocampal regions, suggesting that AFE could attenuate the hippocampal injury via the upregulation of autophagy.

The silent information regulator-1 (Sirt-1) is an essential regulator of many biological processes, including cell proliferation, differentiation, senescence, apoptosis, and metabolism [54,55]. It interacts with numerous protein substrates in several signaling pathways, including Wnt, NF-κB, p53, and Notch. Sirt-1 is expressed in both neurons and astrocytes in the CNS. Numerous neurodegenerative illnesses that are accompanied by inflammation show a decrease in Sirt-1 levels. Additionally, a decrease in Sirt-1 expression may increase NF-κB, IL-6, and IL-1β-mediated neuroinflammation and microglial activation [56,57]. In numerous types of neurodegenerative illness brought on by microglial activation, Sirt-1 has neuroprotective benefits [58]. The deacetylation of p53, NF-κB, and FoxOs, respectively, has been shown to suppress apoptosis, inflammation, and oxidative stress, which are all detrimental to neuronal survival [59], and might involve upregulation of Sirt-1 [60]. In the current study, we found downregulation of Sirt-1 in hippocampal regions of the VPA-treated group and its upregulation with AFE. We did suggest the neuroprotective effect for AFE against VPA-induced autism might involve upregulation of Sirt-1 protein.

## 5. Conclusions

We concluded that palm date aqueous fruit extract could be a potential candidate for reducing VPA-induced autistic-like behavioral changes in rats. The potential underlying mechanisms might involve reduction of the brain oxidative stress, inflammation, and apoptosis, and upregulation of the antioxidant genes Nrf2 and HO-1, Sirt-1, and autophagy in the cerebellum and hippocampus.

## Data Availability

All data of the study are available upon request.
